# From clinical phenotypes to molecular stratification: early differential diagnosis of four-repeat tauopathies

**DOI:** 10.3389/fnagi.2026.1801615

**Published:** 2026-06-16

**Authors:** Alexander M. Bernhardt, Günter U. Höglinger, Carla Palleis

**Affiliations:** 1 Neurologische Klinik und Poliklinik, Klinikum der Ludwig-Maximilians-Universität (LMU) München, Munich, Germany; 2 Deutsches Zentrum für Neurodegenerative Erkrankungen e.V. (DZNE), Standort München, Munich, Germany; 3 Munich Cluster for Systems Neurology (SyNergy), Munich, Germany

**Keywords:** biomarkers, corticobasal degeneration, neurodegenaration, Parkinson’s disease, progressive supra nuclear palsy, synuclein alpha (SNCA), tauopathies

## Abstract

Progressive supranuclear palsy (PSP) and corticobasal degeneration (CBD) are primary tauopathies defined by aggregation of four-repeat (4R) tau, yet early *in vivo* diagnosis remains limited by the dissociation between clinical phenotype and molecular pathology. Clinical presentations are heterogeneous, evolve over time, and frequently overlap with Alzheimer’s disease, synucleinopathies, and mixed pathologies, particularly in corticobasal syndrome. As a result, clinical criteria provide structured phenotypic classification but have constrained specificity in early disease. This review synthesizes current evidence relevant to early diagnostic stratification in 4R tauopathies, integrating clinical criteria, supportive biomarkers of neurodegeneration, and emerging tau-directed molecular tools. The probabilistic value and limitations of contemporary criteria frameworks are discussed alongside the role of structural and functional imaging, dopaminergic imaging, and fluid markers as indicators of disease intensity and progression rather than molecular specificity. Advances in tau positron emission tomography and tau seed amplification assays are reviewed as biologically grounded approaches that directly interrogate aggregated and seed-competent tau species, with growing evidence for their potential contribution to individual-level stratification. Collectively, the literature supports a layered diagnostic approach in which clinical phenotype, supportive biomarkers, and tau-directed molecular measures are integrated to refine attribution of 4R tau pathology *in vivo*, a prerequisite for mechanism-based therapeutic development.

## 1 Introduction

Progressive supranuclear palsy (PSP) and corticobasal degeneration (CBD) are histopathologically defined primary tauopathies with predominant aggregation of 4-repeat (4R) tau isoforms. PSP and CBD are defined by characteristic lesion profiles [e.g., tufted astrocytes in PSP (Hauw et al., 1994; Roemer et al., 2022); astrocytic plaques and ballooned neurons in CBD (Dickson et al., 2002)], with PSP predominantly affecting the basal ganglia and brainstem and CBD more often involving cortical regions. However, their *in vivo* recognition remains constrained by the absence of validated, widely available, pathology-specific biomarkers in routine care. As a result, clinical diagnosis is anchored in syndromic criteria (Armstrong et al., 2013; Höglinger et al., 2017a) that acknowledge broad phenotypic heterogeneity and evolve over time rather than providing early, definitive molecular confirmation.

Early disease is particularly challenging because presentations are incomplete, overlapping, and frequently compatible with alternative neurodegenerative substrates. The diagnostic delay in 4R tauopathies remains substantial, with an average interval of approximately 3–4 years from first symptom onset to diagnosis (Respondek et al., 2014). This delays both timely symptomatic management and potential inclusion in clinical trials. This issue is most explicit in corticobasal syndrome (CBS), which is a clinically defined syndrome characterized by progressive cortical and basal ganglia dysfunction and can arise from diverse underlying pathologies. While CBS is commonly linked to tau-predominant pathologies such as PSP and CBD, Alzheimer’s disease (AD) with β-amyloid pathology and mixed 3R/4R tau pathology, as well as Lewy-type α-synucleinopathies (LTS), TDP-43 proteinopathies, and mixed pathologies, may also underlie the same clinical phenotype (Lee et al., 2011; Ling et al., 2010; Palleis et al., 2025a; Wadia and Lang, 2007). Accordingly, CBD refers to a distinct histopathological entity and should not be equated with CBS, and early clinic-based labels risk systematic misclassification without biomarker support.

**FIGURE 1:**
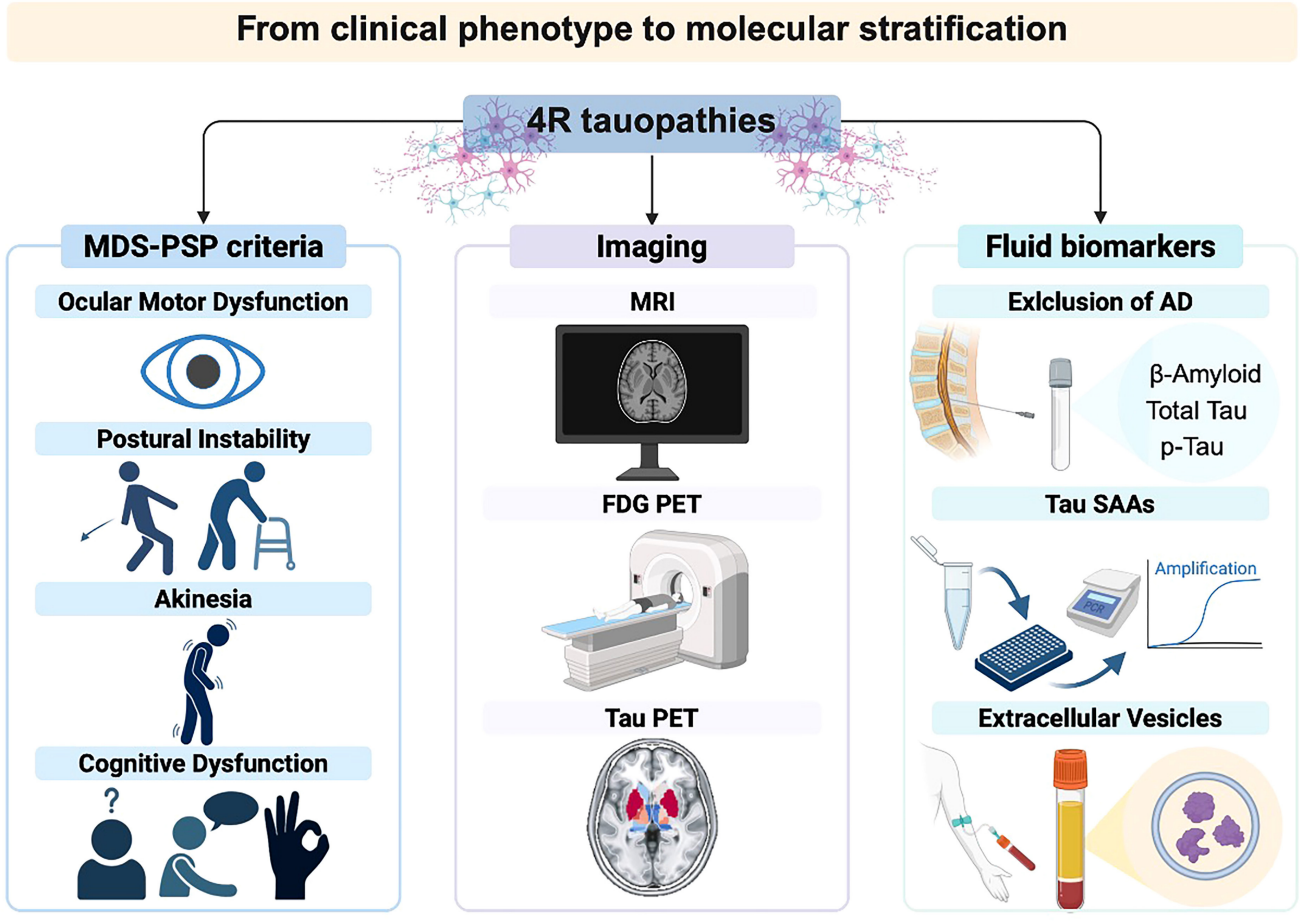
Clinical diagnosis is anchored in the MDS-PSP criteria. Imaging biomarkers include structural MRI, FDG-PET, and tau PET as supportive markers of neurodegeneration and network dysfunction. Fluid biomarkers for molecular stratification include CSF biomarkers for exclusion of Alzheimer’s disease pathology, tau seed amplification assays (SAAs), and plasma-derived extracellular vesicles (EVs). Tau PET, SAAs, and EV-based biomarkers are currently restricted to clinical research settings. Created with BioRender.com.

Current criteria frameworks have improved late-stage diagnostic specificity and provided a structured language for phenotypic variants, but they remain limited in early disease (Ali et al., 2019; Höglinger et al., 2017a), when diagnostic probabilities are most malleable and when emerging disease-modifying strategies would benefit most from accurate substrate identification. This creates a practical need for structured early diagnostic strategies and for biologically grounded positive biomarkers that enable molecular stratification during life. Figure 1 summarizes this layered diagnostic framework, illustrating how clinical phenotype, supportive imaging, and fluid biomarkers are integrated to refine early *in vivo* attribution of 4R tau pathology.

## 2 Clinical heterogeneity and its diagnostic implications

In 4R tauopathies, clinical phenotype is an imperfect proxy for histopathology because syndrome expression reflects the interaction of lesion topography, network vulnerability, and disease stage rather than tau isoform status alone. In PSP, clinicopathological series and longitudinal observational cohorts demonstrate a broad phenotypic spectrum during life, with the classical Richardson syndrome (RS) representing only a subset (Höglinger et al., 2017a). Non-RS variants may initially mimic idiopathic Parkinson’s disease, other atypical parkinsonian syndromes, frontotemporal syndromes, primary progressive aphasia/speech-language phenotypes, or present with corticobasal features, with partial phenotypic convergence later in the disease course (Jabbari et al., 2020; Respondek et al., 2014). This temporal instability means that early clinical labels should be treated as probabilistic signals that modulate pretest probability, not as deterministic entities that confirm PSP biology.

Similarly, CBD is a histopathological entity, whereas CBS is a clinically defined syndrome characterized by progressive cortical and basal ganglia dysfunction and can arise from diverse underlying pathologies, most commonly tau-predominant pathologies (PSP and CBD) or AD, with additional contributions from LTS, TDP-43 proteinopathies, and mixed pathologies (Constantinides et al., 2022; Lee et al., 2011; Ling et al., 2010; Palleis et al., 2025a; Wadia and Lang, 2007). Autopsy series and biomarker-defined *in vivo* cohorts converge on this polyetiology, implying that syndromic recognition of CBS—particularly in early disease—has intrinsically limited positive predictive value for any single substrate. Heterogeneity is further amplified by disease stage: early disease is defined by partial and evolving feature constellations, whereas later disease accrues higher specificity through the emergence of high-weight signs and convergent supportive patterns (Höglinger et al., 2017a). Hence, *late specificity ≠ early sensitivity*: clinical phenotypes become more discriminative over time, but the window in which substrate identification would most strongly shape prognostication, counseling, and trial enrollment is precisely when phenotype is least stable and most confounded by alternative and co-pathologies. Methodologically, phenotype should therefore be treated as an evidence-weighting device defining pretest probability by syndrome, context, and time since onset—rather than as a surrogate for pathology and used to guide the selection of exclusionary and positive biomarkers capable of shifting posterior probability toward or away from AD, synucleinopathy, or 4R tauopathy biology.

The Movement Disorders Society (MDS)-PSP criteria (Höglinger et al., 2017a) represent a conceptual advance by explicitly decoupling clinical phenotype from assumed pathology and formalizing diagnostic reasoning as a domain-based, probabilistic process. Rather than privileging a single “core” presentation, the criteria operationalize PSP as a constellation of abnormalities across four functional domains—ocular motor dysfunction, postural instability, akinesia, and cognitive dysfunction (OPAC) —with graded diagnostic certainty reflecting the weight and temporal emergence of domain-specific features. The introduction of a *“suggestive of PSP”* category is particularly relevant to early disease, as it acknowledges that initial clinical presentations often lack high-specificity features, yet may already carry meaningful risk of underlying 4R tauopathy (Grimm et al., 2020; Quattrone et al., 2025).

Within this framework, CBS is explicitly positioned as a cognitive-domain phenotype. In the MDS-PSP criteria, CBS is mapped to the OPAC domain of cognitive dysfunction, such that the combination of CBS features with vertical supranuclear gaze palsy or slowed vertical saccades permits classification as PSP-CBS. Conceptually, this construct reflects a probabilistic inference: after exclusion of AD, the most likely underlying substrate is a 4R tauopathy, most commonly PSP or CBD. Accordingly, PSP-CBS can be operationalized as a *probable 4R tauopathy* at the syndromic level, while still acknowledging that definitive pathological discrimination between PSP and CBD is not achievable *in vivo* on clinical grounds alone. The Armstrong CBD criteria (Armstrong et al., 2013) likewise recognize CBS as a syndrome with limited pathological specificity, and both PSP and CBD frameworks therefore rely on exclusion logic, most prominently exclusion of Alzheimer’s disease, to maintain diagnostic coherence. Even when rigorously applied, early CBS remains biologically ambiguous by design: the same clinical constellation may reflect PSP, CBD, AD, synucleinopathy, TDP-43 pathology, or mixed disease (Lee et al., 2011; Ling et al., 2010; Wadia and Lang, 2007).

## 3 Early differential diagnosis: exclusion before confirmation

Early presentations compatible with PSP-spectrum disease or CBS rarely provide sufficient positive specificity for a 4R tauopathy. Diagnostic reasoning therefore follows a staged approach: (i) identify the clinical syndrome, (ii) quantify pretest probability by syndrome and context, and (iii) apply molecular biomarkers that meaningfully shift posterior probability toward or away from AD, synucleinopathy, or 4R tauopathy.

### 3.1 Excluding Alzheimer’s disease in corticobasal syndrome

CBS is a clinical syndrome with established clinicopathological heterogeneity; AD pathology is a frequent and legitimate substrate of CBS, alongside CBD, PSP, and less commonly TDP-43 proteinopathies. Consequently, the first high-impact branchpoint in CBS is the presence of AD biology.

Both major criteria frameworks explicitly encode this step. The MDS-PSP criteria require, specifically in PSP-CBS presentations, exclusion of AD pathology by an AD-typical CSF profile (reduced Aβ42 with elevated total/phospho-tau) or amyloid PET positivity [context-dependent exclusion criterion B3 (Jack et al., 2018; Jack et al., 2024; Palleis et al., 2025a)]. The Armstrong CBD criteria similarly treat AD biomarker evidence (amyloid imaging or CSF Aβ42/tau ratio consistent with AD) as an exclusion criterion for CBD categories, reflecting that CBS phenotypes frequently arise from non-CBD pathology and that AD-like presentations would otherwise inflate false-positive CBD labeling.

### 3.2 Distinguishing 4R tauopathies from synucleinopathies

In early akinetic-rigid parkinsonism (including early PSP-P/PSP-PI–like presentations) and in CBS with prominent parkinsonism, the competing alternative diagnosis is a synucleinopathy [Parkinson’s Disease (PD), Dementia with Lewy-Bodies (LBD)and Multiple Systems Atrophy (MSA)]. Clinical enrichment features (e.g., prominent dysautonomia, hallucinations/fluctuations, rapid eye movement sleep behavior disorder) increase pretest probability for synucleinopathy and are incorporated as exclusionary logic in both PSP (Höglinger et al., 2017a) and CBD (Armstrong et al., 2013) clinical criteria, but none are individually definitive in early disease.

α-Synuclein Seed Amplification Assays (αSyn SAA) provide pathology-linked evidence of LTS (Martinez-Valbuena et al., 2025a). However, neuropathology demonstrates that mixed proteinopathies are common in PSP (Jecmenica Lukic et al., 2020) and present in a subset of CBD (Lee et al., 2011; Ling et al., 2010; Wadia and Lang, 2007), including LTS, and AD-related changes are frequent co-pathology in PSP. Across published CBS cohorts, αSyn SAA positivity ranges from ∼3–36 to 35.9% (14/39) in Aβ− CBS in Anastassiadis et al. (2024); in the Amprion SAA cohort 46% (6/13) in AD-CBS and 19% (3/16) in non-AD/unknown-status CBS (Vaughan et al., 2024); and 3.4% (1/29) in Baiardi et al. (2022). In PSP, reported αSyn SAA positivity likewise varies [28.6% (8/28) in Anastassiadis et al., 2024; 10.2% (6/59) in PROSPECT-UK; Vaughan et al., 2024], consistent with variable mixed pathology. Accordingly, αSyn SAA is best interpreted as a probabilistic modifier. Positivity supports the presence of LTS seeding activity, whether primary or co-pathology. Negativity reduces, but does not exclude, LTS, particularly when anatomically restricted (Bernhardt et al., 2025; Levin et al., 2024). Results should therefore be interpreted together with amyloid status and syndrome-defined pretest probability.

## 4 Supportive biomarkers of neurodegeneration and network dysfunction

### 4.1 Imaging biomarkers

Structural MRI remains the most informative imaging modality in the early evaluation of suspected 4R tauopathies, primarily to support probabilistic differentiation and exclude alternative diagnoses rather than to establish definitive pathology. In PSP, characteristic patterns include predominant midbrain atrophy with relative pontine preservation, resulting in the “hummingbird” or “penguin” sign on sagittal imaging (Kaasinen et al., 2015; Oba et al., 2005). Quantitative indices such as the midbrain-to-pons area ratio and the Magnetic Resonance Parkinsonism Index (MRPI), including MRPI 2.0 (Massey et al., 2013; Quattrone et al., 2018; Quattrone et al., 2022), improve discrimination between PSP and PD. In selected cohorts – particularly for PSP-RS and PSP-P versus PD – reported diagnostic performance often exceeds 80–90% sensitivity and specificity., although accuracy is reduced in early disease stages and in non-RS phenotypes. However, subsequent studies highlighted important limitations, including overlap with other atypical parkinsonian syndromes, variability across imaging protocols and cohorts, and inconsistent additional benefit of MRPI 2.0 over conventional MRPI in real-world clinical settings (Kim et al., 2022; Madetko et al., 2022). Accordingly, these markers should be regarded as supportive rather than definitive diagnostic tools. There is evidence suggesting that longitudinal structural MRI may be used as progression marker with atrophy becoming increasingly pronounced over time (Quattrone et al., 2024; Scotton et al., 2023). Atrophy has further been shown to be linked to worsening of symptoms (Dutt et al., 2016; Höglinger et al., 2017b; Picillo et al., 2020) and subcortical atrophy may serve as a predictor of survival in PSP (Whiteside et al., 2023).

In CBS, structural MRI findings are more heterogeneous and reflect the underlying pathological diversity (Di Stasio et al., 2019; Whitwell et al., 2010). Asymmetric frontoparietal cortical atrophy is common but nonspecific and may be observed in CBS due to AD, CBD, PSP, or TDP-43 pathology. Imaging therefore does not reliably distinguish CBS-CBD from CBS-AD or CBS-PSP in early disease stages. Consequently, structural MRI in CBS is primarily used to identify asymmetry patterns, exclude vascular or inflammatory mimics, and guide downstream biomarker testing. There is limited evidence on longitudinal MRI data in CBS. In one study, over 6 and 12 months, there were no significant voxel-wise differences of atrophy between 33 CBS and 55 PSP patients, with baseline atrophy being more severe and widespread in CBS than in PSP (Dutt et al., 2016). In a small study of 21 patients with CBS with probable 4R tauopathy, a prediction modeling revealed that gray matter atrophy was not associated with clinical decline over a follow-up time of approximately 2 years (Palleis et al., 2024).

[^18^F]FDG-PET provides complementary functional information, with hypometabolism patterns that often parallel structural changes. In PSP, frontal and midbrain hypometabolism may be observed, whereas CBS typically shows asymmetric frontoparietal hypometabolism (Buchert et al., 2023; Martí-Andrés et al., 2020). However, FDG-PET reflects network dysfunction rather than molecular pathology and lacks specificity for tau isoform or protein class.

Beyond regional readouts, network-based approaches combine longitudinal structural MRI with resting state functional MRI connectomics to model how neurodegeneration distributes across the brain in PSP (Palleis et al., 2025b; Spinelli et al., 2024). In PSP-RS, regions showing severe baseline atrophy or rapid volume loss preferentially cluster within strongly interconnected networks, and connectivity profiles of individual “atrophy epicenters” can predict patient-level patterns of subsequent atrophy. Such connectome-informed MRI models are not pathology-specific, but they provide a framework for staging and prognostic modeling and may help define sensitive progression readouts for clinical trials.

Dopaminergic imaging using dopamine transporter single-photon emission computed tomography (DAT-SPECT) frequently demonstrates presynaptic striatal dopaminergic deficits in both PSP and CBS. While abnormal DAT-SPECT supports the presence of a neurodegenerative parkinsonian syndrome, it lacks specificity and cannot differentiate 4R tauopathies from synucleinopathies (Bega et al., 2021).

### 4.2 Fluid biomarkers

Neurofilament light chain (NfL) reflects axonal injury and serves as a sensitive but nonspecific marker of neurodegeneration (Khalil et al., 2024). Elevated NfL levels correlate with disease severity, progression rate, and prognosis in PSP and CBS (Donker Kaat et al., 2018; Palleis et al., 2024; Palleis et al., 2025a; Rojas et al., 2016), but similar elevations are observed across atypical parkinsonian syndromes, including MSA, as well as in other neurological conditions (Bridel et al., 2019). Importantly, CSF and blood NfL levels are substantially higher in atypical parkinsonian syndromes than in PD, supporting its utility in distinguishing PD from atypical parkinsonism at early disease stages. Accordingly, NfL is informative for disease intensity and progression, and may aid in differentiating PD from atypical parkinsonian syndromes but lacks discriminatory value for early differential diagnosis of 4R tauopathies.

Increasing attention has recently focused on inflammatory and neurotrophic biomarkers as potential tools for phenotypic characterization in 4R tauopathies (Alster et al., 2020). Differences in serum and CSF concentrations of interleukin-1β (IL-1β) and interleukin-6 (IL-6) have been reported between PSP phenotypes, with lower interleukin levels observed in PSP-RS compared with PSP-P, supporting the hypothesis of subtype-specific neuroinflammatory mechanisms (Madetko-Alster et al., 2023). Further, inflammatory markers such as neutrophil-to-lymphocyte ratio and platelet-to-lymphocyte ratio may be able to reflect cognitive decline in PSP-P and PSP-RS patients, while IL-1β may play a protective role in cognitive function (Chunowski et al., 2024). Iron-regulatory and neurotrophic pathways may also contribute to phenotypic heterogeneity, as altered hepcidin and glial cell line-derived neurotrophic factor (GDNF) levels were reported in PSP-RS and PSP-P (Alster et al., 2024a; Alster et al., 2024b). Recent high-sensitivity multiplex proteomic analyses using the nucleic acid linked immuno-sandwich assay (NULISA) platform identified peripheral inflammatory signatures in PSP, highlighting the potential of inflammatory profiling for future biomarker development (Durcan et al., 2025). Nevertheless, these findings on inflammatory and neurotrophic markers in 4R tauopathies remain preliminary and require validation in larger longitudinal and neuropathologically confirmed cohorts before clinical implementation.

## 5 Promising emerging molecular diagnostics in 4R tauopathies

Despite advances in multimodal biomarkers, reliable *in vivo* molecular discrimination between PSP and CBD remains limited. Current approaches primarily support identification of a probable underlying 4R tauopathy rather than disease-specific classification, and should therefore be interpreted within a probabilistic framework.

The interpretation of emerging molecular biomarkers in 4R tauopathies requires consideration of their stage of development and supporting evidence. Biomarkers can be broadly stratified into proof-of-concept, early replication-stage, and near-clinical tools with established analytical and clinical validity (Bernhardt et al., 2023). Within this framework, tau PET represents an advanced but still evolving modality with growing multicenter evidence, αSyn SAAs approach near-clinical readiness, whereas CSF-based tau SAA and extracellular vesicle (EV)-based biomarkers remain at a proof-of-concept stage, and peripheral tau SAA approaches (e.g., skin-based assays) can be considered early replication-stage tools requiring further validation.

### 5.1 Tau PET imaging in 4R tauopathies

Tau PET is biologically motivated as an *in vivo* measure of aggregated tau and, in PSP and CBS, is increasingly applied as a pattern-based and distribution-sensitive biomarker that reflects disease-relevant tau burden. Ligand binding characteristics, regional vulnerability, and cellular substrates vary across tauopathies and tracers (Beyer and Brendel, 2021). Converging evidence indicates that tau PET can reliably distinguish AD-typical neocortical 3R/4R tau distributions—most often in Aβ-positive patients—from non–AD-like patterns characterized by predominant subcortical and basal ganglia involvement that are consistent with 4R tauopathy biology, while acknowledging that neither pattern is fully isoform-specific.

First-generation flortaucipir ([^18^F]AV-1451) is strongly validated for AD-type tau (Passamonti et al., 2017) but has shown limited and inconsistent utility in 4R tauopathies, where signal is generally weaker and more vulnerable to substantial off-target binding and methodological confounds, including binding to neuromelanin, microhemorrhages, choroid plexus and monoamine oxidase-B (Ishiki et al., 2018; Marquié et al., 2017a; Marquié et al., 2017b). Consequently, [^18^F]AV-1451 is not considered a reliable tool to establish 4R tau pathology and is better viewed as an AD-focused tracer.

Next-generation ligands, particularly [^18^F]PI-2620, [^18^F]PM-PBB3 and more recently [^18^F]Florzolotau, have produced more encouraging signals in 4R tauopathies (Mena et al., 2023), showing affinity both for AD-type 3-repeat/4-repeat tau as well as 4R tau (Brendel et al., 2020; Brumberg et al., 2025; Hong et al., 2023; Lin et al., 2026; Liu et al., 2025; Palleis et al., 2021; Shimohama et al., 2023; Tagai et al., 2021). Although these tau tracers demonstrate substantially reduced off-target binding compared with first-generation ligands, nonspecific signal retention and background uptake in subcortical regions still require cautious interpretation in 4R tauopathies (Aguero et al., 2024). Direct head-to-head evidence of the next-generation PET tracers [^3^H]PI-2620, [^3^H]MK-6240 and [^3^H]-RO948 indicates that different tracers may diverge substantially in a small study of frontal lobe tissue from deceased patients with PSP (*n* = 3) or CBD (*n* = 2) pathology under specific experimental conditions (Malarte et al., 2023), but these findings require replication in larger cohorts to confirm their generalizability. For [^18^F]PI-2620, multiple *in vivo* studies support the ability to detect group-level differences consistent with PSP/CBS-spectrum tau topographies and to support classification workflows that emphasize distributional reads (Bauer et al., 2025; Blazhenets et al., 2023; Brendel et al., 2020; Messerschmidt et al., 2022; Palleis et al., 2021), e.g., detection of PSP-RS vs. healthy controls with 85% sensitivity in a prospective multicenter study (Brendel et al., 2020). For [^18^F]Florzolotau, a single-center study using visual scale methods and decision tree analyses has reported sensitivities of 89.7–100% and specificities of 89.7–96.7% to distinguish AD, PSP, and healthy controls (Lin et al., 2026). However, these findings remain to be replicated in formal multicenter cohorts, which is important for confirming reproducibility and broader clinical applicability. [^18^F]PI-2620 tracer’s binding characteristics further facilitate discrimination between the tau isoforms through evaluation of region-specific distribution volume ratios, perfusion patterns and tracer tissue clearance (Beyer et al., 2020; Katzdobler et al., 2023; Song et al., 2021a; Song et al., 2021b). The combination of CSF biomarkers (p-tau181, t-tau) and [^18^F]PI-2620 tau-PET may further provide added value by enabling more accurate differentiation between AD and 4R-tauopathies (Dilcher et al., 2024). Integration of [^18^F]PI-2620 PET with resting-state fMRI indicates that functionally connected regions show correlated tau deposition in 4R tauopathy patients, driven by neuronal tau (Franzmeier et al., 2022). This is further supported by translational data that suggest that [^18^F]PI-2620 PET signal may be substrate- and cell-type dependent, with comparatively stronger correspondence to neuronal fibrillar tau than to astroglial-predominant lesions (Slemann et al., 2024), which is mechanistically relevant in 4R tauopathies. There is limited evidence on tau-PET as a prediction or monitoring marker. Our group investigated in a cohort of 21 CBS patients with diagnosis of probable 4R tauopathy (Höglinger et al., 2017a) that [^18^F]PI-2620 tau-PET tracer uptake is associated with clinical decline in these CBS patients, underlining its potential as a stratification tool for clinical decision-making and clinical trials (Palleis et al., 2024). Further, first longitudinal data indicate potential utility for monitoring change in selected cohorts, but also raise the possibility of stage-dependent dynamic range limitations (including apparent plateauing/saturation in advanced disease), which can weaken severity correlations (Gnörich et al., 2026).

Across tracers, tau PET shows increasing promise in PSP/CBS, with remaining challenges related to off-target binding, methodological heterogeneity, and differential sensitivity to tau conformers and cellular substrates. Ongoing standardization efforts and expanding pathology-validated datasets are expected to further improve its reliability for biological stratification and longitudinal assessment in 4R tauopathies.

### 5.2 Tau seeding assays

Tau SAAs provide a mechanistically distinct biomarker class by directly exploiting the prion-like templated propagation of misfolded tau, thereby targeting the disease-defining protein species rather than downstream neurodegeneration. In contrast to ligand-based imaging, tau SAAs are agnostic to regional vulnerability and tracer affinity and instead probe the intrinsic seeding competence and conformational properties of aggregated tau.

Brain-based work established assays capable of selectively amplifying 4R tau seeds from PSP and CBD tissue with multi-log sensitivity and strong discrimination from 3R or mixed 3R/4R tauopathies (Saijo et al., 2020). A key conceptual advance was the demonstration that amplified products preserve disease-associated conformational features, allowing subclassification of 4R tau seeds into distinct templating “classes,” consistent with molecular heterogeneity within and across PSP and CBD. Importantly, published CSF tau SAA data have not reached the level of independent replication and diagnostic performance seen with α-Synuclein SAAs, and current results should therefore be viewed as proof-of-concept rather than clinically deployable biomarkers.

More recently, peripheral detection has expanded the biological scope of tau SAAs (Martinez-Valbuena et al., 2024; Wang et al., 2024). Martinez-Valbuena et al. (2025b) reported 4R tau seeding activity in skin biopsies from patients with PSP, providing initial evidence that seed-competent tau aggregates are detectable outside the central nervous system. Skin-based SAAs are biologically appealing because they combine minimally invasive sampling with direct detection of pathogenic tau conformers, enabling repeatable longitudinal assessment. However, available data are preliminary, with open questions regarding anatomical sampling sites, phenotype dependence, assay standardization, sensitivity in early disease, and specificity across 4R tauopathies.

### 5.3 Extracellular vesicles

Extracellular vesicles (EVs) have emerged as promising biomarkers of tau pathology. They are released by various brain cell types into the extracellular space and peripheral circulation, enabling minimally invasive access to brain-derived molecular information (Ho, 2025). In contrast to other biofluids, EVs contain a substantial fraction of full-length tau, allowing for the reliable detection of distinct tau isoforms (Chatterjee et al., 2024). Beyond their biomarker potential, EVs are also implicated in the intercellular transport and propagation of pathological tau aggregates, supported by evidence that aggregated proteins are preferentially sorted into EVs (Fowler et al., 2025; Shen et al., 2011). First promising analyses of large clinical cohorts demonstrated that plasma EV-associated tau isoforms may have the ability to distinguish PSP from healthy controls as well as from other neurodegenerative disorders, underscoring their diagnostic relevance (Chatterjee et al., 2024). Importantly, EVs may constitute a patient-friendly biomarker source, given their accessibility through minimally invasive blood-based assays.

## 6 Discussion and future directions

Early diagnosis in 4R tauopathies is constrained by a dissociation between clinical expression and molecular pathology. Clinical syndromes reflect lesion distribution, network vulnerability, and disease stage, whereas pathology is defined by protein species, conformation, and cellular substrate. This mismatch explains why diagnostic specificity increases late in the disease course, while early sensitivity remains limited, and why early diagnostic confidence cannot be achieved through clinical criteria alone.

Across PSP and CBS, diagnostic reasoning therefore proceeds by staged probabilistic inference. In CBS in particular, exclusion of Alzheimer’s disease represents the most decisive early branchpoint, as amyloid positivity strongly supports an AD biological substrate, whereas amyloid negativity increases the likelihood of a 4R tauopathy once alternative causes are excluded (Armstrong et al., 2013; Jack et al., 2024).

The MDS-PSP criteria (Höglinger et al., 2017a) provide an essential conceptual framework for early diagnostic reasoning by formalizing phenotype as a probabilistic, domain-based signal rather than a categorical diagnosis. The “suggestive of PSP” category explicitly acknowledges early uncertainty and aligns clinical classification with staged inference. Nevertheless, even when rigorously applied, criteria cannot discriminate between PSP, CBD, and alternative substrates in early disease (Grimm et al., 2020; Quattrone et al., 2025). Especially CBS is intrinsically polyetiological, and early PSP phenotypes frequently overlap with synucleinopathies, AD-spectrum disease, and mixed pathologies (Jabbari et al., 2020; Lee et al., 2011; Ling et al., 2010; Wadia and Lang, 2007).

Imaging and fluid biomarkers therefore function best as complementary probability modifiers rather than standalone arbiters of pathology. Structural MRI, FDG-PET, and DAT-SPECT support syndrome-level differentiation and exclusion of mimics but lack molecular specificity. Tau PET has demonstrated encouraging performance at the individual level in selected cohorts and supports biological stratification by regional distribution and substrate context. However, tracer-dependent binding properties, off-target effects, and variable sensitivity to astroglial-predominant tau pathology currently limit universal diagnostic applicability. Tau SAAs offer a biologically grounded alternative by directly interrogating seed-competent tau species, yet translation to lumbar CSF and peripheral tissues remains preliminary, with unresolved questions regarding sensitivity, specificity, and inter-laboratory robustness. Despite the promising first results on EV-based tau biomarkers in plasma, evidence on EV biomarkers remains limited, as these observations await independent confirmation from other laboratories. Beyond protein aggregation, additional mechanisms—including neuroinflammatory processes and associated metabolic pathways such as the kynurenine system (Chakraborty et al., 2026; Taj et al., 2025) —may contribute to disease heterogeneity and biomarker variability. Furthermore, integrative approaches combining imaging and fluid biomarkers represent a promising strategy to capture the complexity of early disease detection (Chakraborty et al., 2025; Das et al., 2025).

Collectively, available evidence supports a layered diagnostic strategy in which clinical phenotype defines pretest probability, high-yield exclusionary biomarkers (amyloid and α-synuclein) perform early branching, and tau-directed biomarkers increasingly refine 4R tauopathy attribution. Ultimately, the field requires validated molecular biomarkers that identify the relevant pathogenic species *in vivo*. Disease-modifying therapies will be directed at specific molecular targets and therefore require mechanism-based eligibility and target engagement readouts. Translation into clinical trials and routine care requires not only analytical and clinical validation but also formal qualification within a defined context of use, for example as a diagnostic, prognostic, predictive, or safety biomarker, which determines evidentiary requirements and clinical applicability, as outlined by regulatory frameworks (e.g., the U.S. Food and Drug Administration and European Medicines Agency biomarker qualification programs). In addition, harmonization of acquisition protocols, inter-laboratory reproducibility, and multicenter validation remain critical prerequisites for the use of biomarkers as stratification and target engagement tools.
